# A Differential Redox Regulation of the Pathways Metabolizing Glyceraldehyde-3-Phosphate Tunes the Production of Reducing Power in the Cytosol of Plant Cells

**DOI:** 10.3390/ijms14048073

**Published:** 2013-04-12

**Authors:** Claudia V. Piattoni, Sergio A. Guerrero, Alberto A. Iglesias

**Affiliations:** Instituto de Agrobiotecnología del Litoral (IAL, CONICET-UNL), FBCB, Paraje “El Pozo”, CC 242, Santa Fe S3000ZAA, Argentina; E-Mails: piattoni@fbcb.unl.edu.ar (C.V.P.); sguerrer@fbcb.unl.edu.ar (S.A.G.)

**Keywords:** plant glycolysis, redox regulation, NADPH generation, ATP production, triose-phosphate dehydrogenase

## Abstract

Adaptation to aerobic life leads organisms to sense reactive oxygen species and use the signal for coordination of the entire metabolism. Glycolysis in plants is a particular network where specific steps, like oxidation of glyceraldehydes-3-phosphate (Ga3P), are critical in order for it to function. The triose-phosphate can be converted into 3-phosphoglycerate through the phosphorylating Ga3P dehydrogenase (Ga3PDHase, EC 1.2.1.12) producing ATP and NADH, or via the non-phosphorylating enzyme (*np*-Ga3PDHase; EC 1.2.1.9) generating NADPH. In this work we found redox regulation to be a posttranslational mechanism allowing the fine-tuning of the triose-phosphate fate. Both enzymes were inactivated after oxidation by reactive oxygen and nitrogen species. Kinetic studies determined that Ga3PDHase is marked (63-fold) more sensitive to oxidants than *np*-Ga3PDHase. Thioredoxin-*h* reverted the oxidation of both enzymes (although with differences between them), suggesting a physiological redox regulation. The results support a metabolic scenario where the cytosolic triose-phosphate dehydrogenases are regulated under changeable redox conditions. This would allow coordinate production of NADPH or ATP through glycolysis, with oxidative signals triggering reducing power synthesis in the cytosol. The NADPH increment would favor antioxidant responses to cope with the oxidative situation, while the thioredoxin system would positively feedback NADPH production by maintaining *np*-Ga3PDHase at its reduced active state.

## 1. Introduction

The glycolytic pathway is a catabolic process of high ubiquity between living cells and occupying a position of historical and philosophical importance in biochemistry. The characterization of glycolytic enzymes strongly contributed to evidence the occurrence of functional cell-free enzymatic processes [1]. Glycolysis, term first applied to describe the fermentation of glucose to lactic acid by muscle tissues, is now used in general to denote the pathway catabolizing sugars to pyruvic acid, which is later metabolized with many variants [1,2]. The so-called classical glycolysis is the cytosolic linear sequence of 10 enzymatic reactions that oxidizes hexoses to generate ATP, NADH, pyruvate, and building blocks for anabolism [2]. This type of glycolysis takes place in most kind of organisms, but in plants the pathway has some distinctive features. In photosynthetic organisms, the glycolytic route oddly occurs in parallel in two separated compartments, the cytosol and the plastids, which are interconnected by specific protein membrane transporters. On the other hand, glycolysis in the cytosol of plant cells is a particularly complex network with alternative enzymatic reactions at critical steps. The latter constitutes bypasses of the main pathway giving flexibility and unique regulatory aspects to the route [1,2]. In plants, glycolysis is the main fuel for respiration, since mitochondria in these organisms use glycolytically generated pyruvate and rarely fatty acids (but predominant in developing oilseeds) as main carbon and energy supplier. The glycolytic metabolism is also a key in plant heterotrophic tissues to deliver the carbon and energy source after the breakdown of starch or incoming sucrose, as these tissues cannot photosynthesize triose-P [2]. Despite many characteristics of the enzymes involved in branch points of the glycolytic route were determined, regulatory mechanisms related to their functioning under different cell situations are still poorly understood.

The present work is centered on the study of two plant cytosolic enzymes mediating alternative pathways for the oxidation of glyceraldehyde-3-phosphate (Ga3P), to 3-phosphoglycerate (3PGA) [1–3]. Through classic glycolysis, the process occurs by two consecutive enzymes [2,3]. First, the reversible NAD^+^-dependent Ga3P dehydrogenase (EC 1.2.1.12, Ga3PDHase) oxidizes Ga3P with coupled phosphorolysis to generate NADH and 1,3-bis-PGA (Reaction I). The latter intermediate, a high-content energy compound, allows phosphorylation at the substrate level (Reaction II) catalyzed by phosphoglycerate kinase (EC 2.7.2.3, PGKase). The alternative pathway is performed irreversibly in one step by oxidation tied to hydrolysis (rather than phosphorolysis) mediated by the non-phosphorylating NADP^+^-dependent Ga3PDHase (EC 1.2.1.9, *np*-Ga3PDHase) to generate NADPH (Reaction III) (see [3] for details). The parallel occurrence of both routes sets out an important difference for cell energetic, since depending on the relative levels of activity of each triose-P DHase the oxidation of Ga3P will provide different amounts of energy (ATP) and/or reducing power (NADPH) in the cytosol [2,3].

(Reaction I) $$\text{Ga}3\text{P}+{\text{NAD}}^{+}+\text{Pi}↔1,3-\text{bis}-\text{PGA}+\text{NADH}+{\text{H}}^{+}$$

(Reaction II) 1,3-bis-PGA+ADP↔3PGA+ATP

(Reaction III) $$\text{Ga}3\text{P}+{\text{NADP}}^{+}+{\text{H}}_{2}\text{O}\to 3\text{PGA}+\text{NADPH}+2{\text{H}}^{+}$$

The physiological function played by *np*-Ga3PDHase in plants and the occurrence of mechanisms regulating its activity have been evidenced [4–8]. An Arabidopsis mutant lacking this enzyme was characterized as having altered morphology of siliques, inhibited glycolytic flux, decreased CO_2_ fixation capacity, and increased sensitivity to oxidative stress [8]. In agreement with the latter, the activity of *np*-Ga3PDHase was found to increase up to 2-fold when wheat and maize seedlings were exposed to oxidative conditions imposed by methylviologen [4]. The effect observed under moderate oxidant concentrations was shown to be a consequence of a significant stability of the enzyme rather than to mRNA induction. On the other hand, *np*-Ga3PDHase was identified as a target for posttranslational phosphorylation in non-photosynthetic plant tissues [5]. The phosphorylated enzyme exhibits distinctive kinetic properties after interaction with 14-3-3 regulatory proteins, becoming less active and more sensitive to regulation by adenylates and inorganic pyrophosphate [6]. Recently [7], we revealed that serine-404 in the wheat *np*-Ga3PDHase is phosphorylated by a member of the SnRK1 (SNF1-related protein kinase) family, a system playing key roles for major integration of energy signaling and growth in plants [9].

Concerning plant Ga3PDHase, its functionality, regulation and involvement in stress situations have also been reported [10–13]. Arabidopsis lines deficient in this enzyme exhibit delayed growth, low number of seeds and siliques with altered morphology [13]. A proteomic approach analysis identified the enzyme as the most prominent protein modified by hydrogen peroxide (H_2_O_2_) in the cytosol of *Arabidopsis thaliana*[11]. Also, *in vitro* studies showed inactivation of the enzyme after oxidation with H_2_O_2_, oxidized glutathione (GSSG), and *S*-nitrosogluthathione (GSNO); being the process reverted by dithiotreitol (DTT) and reduced glutathione (GSH) [11,12]. Recently [10], work performed with the recombinant enzyme from *Arabidopsis* revealed that oxidation of the protein involves formation of sulfenates that can drive the oxidation to an irreversible situation. Albeit, in the presence of GSH Ga3PDHase can reach glutathionylated (inactive) forms, which can be rescued by plant cytosolic glutaredoxin. Interestingly, it was determined that thioredoxin from plants are functional contributors to recover the enzyme from the mixed disulfide state.

Despite the previous findings, studies to comparatively understand the regulation of both cytosolic Ga3PDHases at the molecular level are lacking. In this work, we utilize the enzymes from wheat, produced recombinantly with high purity, to characterize their respective responses to redox modification. We determined that both enzymes are *in vitro* inactivated by oxidation with reactive oxygen and nitrogen species (ROS and RNS, respectively); and they were effectively recovered from the oxidative state by thioredoxin-*h* (TRX-*h*) from wheat. Ga3PDHase was markedly more sensitive to oxidation (also more inert to later reduction) than *np*-Ga3PDHase. Results are analyzed in the metabolic scenario where cytosolic triose-P intermediates are rerouted toward increase synthesis of NADPH under oxidative cell conditions.

## 2. Results

Both wheat triose-P DHases were produced as recombinant enzymes (via heterologous expression in *Escherichia coli*) highly purified (see Supplementary Figure S1) by immobilized metal ion affinity chromatography (IMAC), as was previously described for the *np*-Ga3PDHase [14]. The Ga3PDHase was obtained as a homotetrameric protein (as determined by gel filtration on Superdex 200, Supplementary Figure S2) exhibiting *S*_0.5_ values for NAD^+^ (136 μM) and Ga3P (86 μM) similar to those reported for the enzyme purified from pea shoot and seed [15]; while *V*_max_ (38 U/mg) was found in agreement with that reported for the recombinant Arabidopsis Ga3PDHase [12]. Also, the kinetic and structural properties of the *np*-Ga3PDHase were coincident with the behavior of the enzyme purified from plants, as detailed before [14]. Thus, recombinant production renders both wheat enzymes as proteins that are kinetically and structurally comparable to those obtained from the natural source and consequently accurate for biochemical studies.

**Figure 1:**
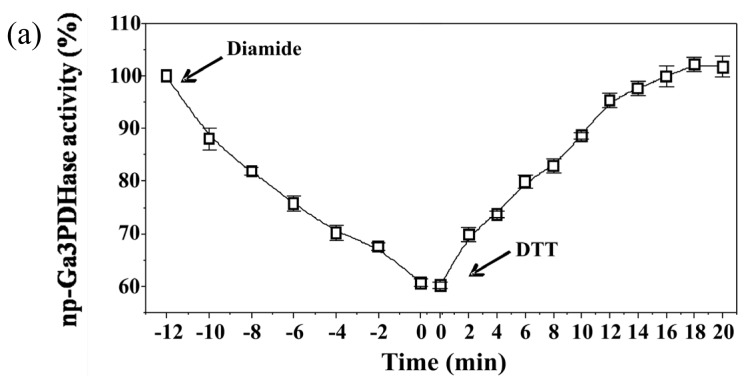

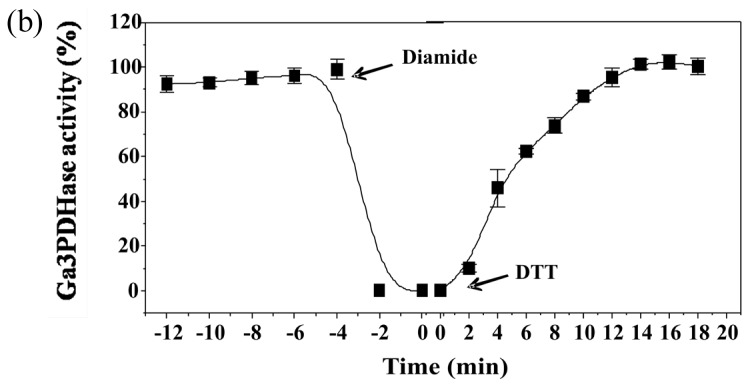
The effect of chemical redox compounds on enzyme activity. (**a**) *np*-Ga3PDHase; (**b**) Ga3PDHase. Each enzyme was incubated as detailed under Experimental Section. When indicated by the arrows it was added 1 mM diamide and then 10 mM dithiotreitol (DTT). At the specified time aliquots were withdrawn and assayed for the respective enzymatic activity. At the beginning of oxidation assays both enzymes were fully active (100%): (**a**) 21 U/mg and (**b**) 38 U/mg. Error bars represent the values deviation in three independent assays.

To analyze the effect of redox agents on *np*-Ga3PDHase and Ga3PDHase we incubated each purified enzyme with the redox chemical agent diamide (oxidant) or DTT (reductant) and determined the activity at different times. Figure 1 shows that diamide inactivated both enzymes, although Ga3PDHase lost its activity more rapidly than *np*-Ga3PDHase. Interestingly, following the addition of DTT both oxidized enzymes recovered the activity (Figure 1), data reinforcing a redox dependence of the proteins’ catalytic capacity. Although neither diamide nor DTT are cellular components, our *in vitro* results suggest that posttranslational modifications by redox mechanisms could be involved in regulating the activity of these enzymes. To further explore about a physiological significance for such a regulation we evaluated the exposure of cytosolic triose-P DHases to redox agents found in plant cells; specifically: (i) the ROS compound H_2_O_2_, (ii) sodium nitroprusside (SNP) which is a donor of the RNS nitric oxide, (iii) glutathione (either GSSG or GSH), and (iv) wheat TRX-*h*.

**Figure 2:**
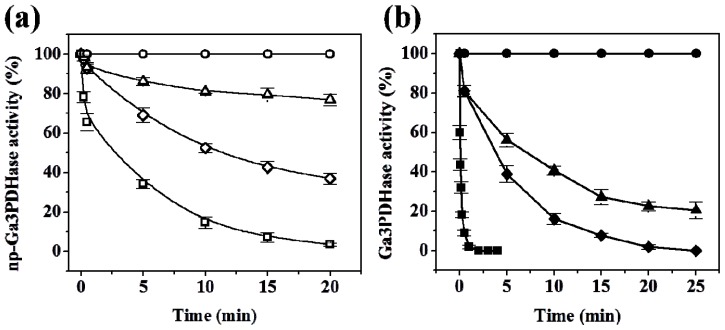
Enzyme inactivation caused by oxidation with H_2_O_2_, nitroprusside (SNP), and GSSG. Progressive inactivation of each triose-P DHase after treatment with the different physiological oxidants as detailed in the Experimental Section. (**a**) Incubation of *np*-Ga3PDHase with: No further addition (○), 3 mM H_2_O_2_ (□), 5 mM SNP (◇) or 20 mM GSSG (Δ). **(b)** Incubation of Ga3PDHase with: No further addition (●), 1 mM H_2_O_2_ (■), 1 mM SNP (◆) or 20 mM GSSG (▲). One hundred percent of activity corresponds to 21 U/mg and 38 U/mg in (**a**) and (**b**), respectively. Error bars represent the values deviation in three independent assays.

As shown in Figure 2, *np*-Ga3PDHase and Ga3PDHase were inactivated to different degree after incubation with the physiological oxidants H_2_O_2_, SNP and GSSG. A first detailed observation indicates that the NAD^+^-dependent (EC 1.2.1.12) enzyme is more sensitive to the oxidants (remarkably toward H_2_O_2_) than the *np*-Ga3PDHase. For a better characterization of the dissimilar sensitivity of the enzymes we performed a kinetic study of the loss of activity caused for each of the oxidants. Inactivation was dependent on time and the concentration of the respective compound, as the oxidative treatments coursed via chemical modification of thiol groups from critical cysteine residues in the respective enzyme. Using the model proposed by Kitz and Wilson [16] for the irreversible inhibition of enzymes (see Experimental Section), we determined the kinetic parameters detailed in Table 1, which evaluate the degree of effectiveness (directly related to the value of *k*”) for each inhibitor as well as the mechanism by which they modify (inactivate) each triose-P DHase. As shown, for both enzymes the effectiveness of the oxidants followed the same order: H_2_O_2_ > SNP > GSSG. From an analysis of Table 1 it is clear that for each oxidant the value of *k*” determined for Ga3PDHase resulted significantly higher than that of the non-phosphorylating enzyme (EC 1.2.1.9), which indicates that the former DHase is more sensitive to oxidation by the respective redox agent. The most outstanding situation is oxidation by H_2_O_2_, where the *k*” value for Ga3PDHase is 63-fold higher than that obtained for *np*-Ga3PDHase. Also, oxidation of the NAD^+^-dependent enzyme by H_2_O_2_ was 26-fold and 700-fold higher than by SNP and GSSG, respectively; whereas *np*-Ga3PDHase oxidation by H_2_O_2_ and SNP were in the same order. GSSG exhibited a very modest effect on the latter enzyme (Table 1). For both enzymes, inactivation caused by H_2_O_2_ coursed in one step, with practically no formation of one stable enzyme-inhibitor intermediate, while for the other two oxidants the mechanism occurred in two steps. Following the model developed by Kitz and Wilson [16] (see details under Experimental Section), oxidation by SNP and GSSG course with a first step where these compounds form a reversible intermediary complex with the enzyme, to follow a second step that leads to the formation of a covalent bond (the disulfide bridge) turning the reaction irreversible.

**Figure 3:**
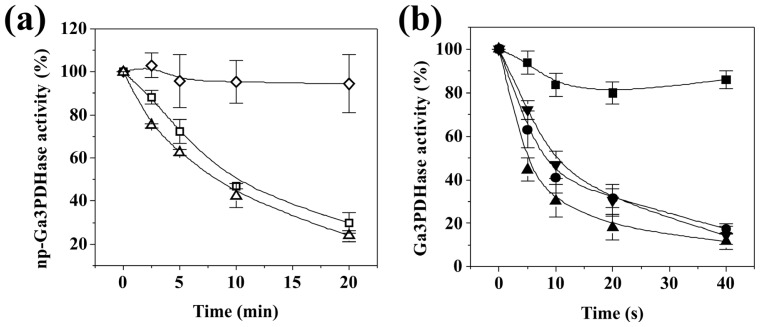
Inactivation of each triose-P DHase by H_2_O_2_ in the presence of substrates. **(a)** Incubation of *np*-Ga3PDHase (100% activity of 21 U/mg) with 1 mM H_2_O_2_ in the presence of none (Δ), 1 mM Ga3P (□) or 100 μM NADP^+^ (◇). Oxidation was followed in time during 20 min at 25 °C; (**b**) Incubation of Ga3PDHase (100% of activity of 38 U/mg) with 0.5 mM H_2_O_2_ in the presence of none (▲), 1 mM NAD^+^ (●), 1.2 mM Ga3P (▼) or 1 mM NAD^+^ plus 1.2 mM Ga3P (■). Oxidation was followed in time during 40 s at 25 °C. Error bars represent the values deviation in three independent assays.

It has been described [3,17–19] that, with same differences, the enzymatic mechanism of Ga3PDHase and *np*-Ga3PDHase comprises the formation of a thiohemiacetal between Ga3P and an essential Cys residue (highly conserved in these enzymes, being Cys^154^ and Cys^298^ those corresponding to the respective wheat protein). The catalytic Cys residues need to be in the reduced state to carry out the catalytic process. To evaluate the possible involvement of these essential Cys residues in the oxidation treatment under study, we tested the effect of the substrates on the inactivation exerted by H_2_O_2_ for both enzymes (Figure 3). The presence of NADP^+^ (but not that of Ga3P) protected *np*-Ga3PDHase against inactivation by the oxidant (Figure 3a). The degree of protection was dependent on the dinucleotide concentration in the modification medium, after which we were able to calculate a *K*_d_ of 2.5 μM for the binding of the substrate (see Experimental Section). In the case of Ga3PDHase, the presence of Ga3P or NAD^+^ separately produce no important change in the modification, but the combined addition of both substrates effectively exerted a protective effect (Figure 3b). The degree of protection in this case was not highly affected by different concentrations of NAD^+^, but it was variable respect to Ga3P in a concentration dependent manner (with a *K*_d_ for the triose-P calculated in 115 μM in the presence of 1 mM NAD^+^).

Results on the protection by substrates agree with the catalytic mechanism described for *np*-Ga3PDHase, where NADP^+^ binds first to the enzyme and induces a local structural re-arrangement making accessible and leaving well positioned the catalytic Cys residue to form a competent thiohemiacetal intermediate [18]. Although NADP^+^ binding was found to expose the catalytic Cys residue, it seems that the conformational change could not favor the reaction of the enzyme with H_2_O_2_, giving no specific insight about the involvement of that Cys residue in the redox regulation mechanism. Concerning the protective effect of Ga3P combined with NAD^+^ to the oxidation of Ga3PDHase, it is in concordance with the catalytic mechanism described for the enzyme from mammals and bacteria [17], where the triose-P forms the thiohemiacetal derivative in the presence of NAD^+^, and suggest that the essential Cys^154^ residue could be at least one of the residues being modified by oxidation. On the other hand, the *K*_d_ values determined for the respective substrate giving protection to inactivation are similar to the corresponding *S*_0.5_ obtained kinetically (detailed above) for each triose-P DHase.

It is widely known that posttranslational regulation by redox mechanisms comprises the activation or inactivation of enzyme activity by the occurrence of reversible oxidation-reduction reactions [9,20,21]. In order to test if the oxidation of Ga3PDHase and/or *np*-Ga3PDHase by H_2_O_2_ is possible to be reverted by reducing physiological components, we incubated the oxidized enzymes with variable concentrations of recombinant reduced TRX-*h* and followed the reaction by enzyme activity assays.

**Figure 4:**
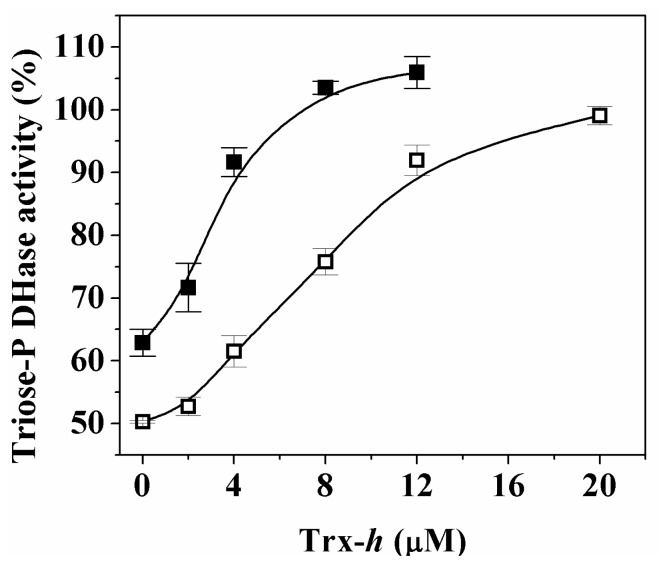
Effect of thioredoxin-*h* (TRX-*h*) on inactive triose-P DHases. *np*-Ga3PDHase (open symbols) was oxidized with 1 mM H_2_O_2_ during 30 min, desalted and then incubated with the stated concentrations of reduce TRX-*h* during 30 min. Ga3PDHase (filled symbols) was oxidized with 0.5 mM H_2_O_2_ during 30 s and the oxidation stopped by addition of catalase (10 U). The enzyme was highly diluted and incubated in the presence of the stated concentrations of TRX-*h* during 2 min. One hundred percent of activity corresponds to 21 U/mg and 38 U/mg for *np*-Ga3PDHase and Ga3PDHase, respectively.

**Figure 5:**
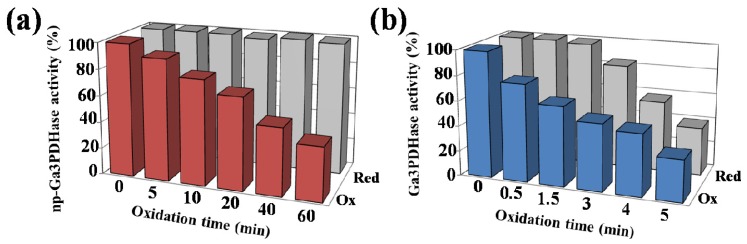
Treatment with TRX-*h* of triose-P DHases inactivated to different degrees. **(a)***np*-Ga3PDHase was oxidized with 1 mM H_2_O_2_ during 0 to 60 min to reach different activity values (shown in % by front bars, Ox). Samples were desalted and then incubated with 20 μM TRX-*h* during 30 min, after which activity was assayed for activity (shown by rear bars, gray); **(b)** Ga3PDHase was oxidized with 0.5 mM H_2_O_2_ during 0 to 5 min to reach different activity values (shown in % by front bars, Ox). After stopping the oxidation by adding catalase (10 U), the respective samples were desalted, incubated with 12 μM TRX-*h* during 30 min and then assayed for activity (shown by rear bars, gray). One hundred percent of activity corresponds to 21 U/mg and 38 U/mg in (**a**) and (**b**), respectively. All assays were repeated at least three times and were reproducible within ± 10%.

Activity of both proteins was completely restored by reductive treatment with increasing concentrations (μM) of reduced TRX-*h* (Figure 4). These results suggest that regulation of both cytosolic triose-P DHases by reversible redox mechanisms could occur *in vivo* under specific circumstances. To further analyze the reduction process, we evaluated the reduction of both enzymes by TRX-*h* after increasing oxidation times (Figure 5). Figure 5a shows that the recovery of activity of *np*-Ga3PDHase by TRX-*h* took place independently of the inactivation degree reached by oxidation. According to Figure 5b the reversion by TRX-*h* is different for Ga3PDHase, as it was effectively exerted on the enzyme inactivated up to a certain level, beyond which the reduction becomes inefficient. It is worth mentioning that, for the Ga3PDHase, similar results were obtained when GSH was the reductant, although the latter was not able to reactivate the *np*-Ga3PDHase (data not shown). In our hands, the effect of TRX-*h* was only in one direction, as its oxidized form had no inhibitory effect on the activity of any of the triose-P DHases under study.

Results showed herein indicate that redox regulation of each triose-P DHase would occur by different mechanisms. Results tempt us to speculate that *np*-Ga3PDHase oxidation would occur by forming of a disulfide bond, which can be reverted by TRX-*h*, as it was previously reported [22]. Using DTNB (see Experimental Section) we quantified 1.8 ± 0.3 and 6.2 ± 0.4 accessible cysteinyl moieties for the oxidized and reduced enzyme, respectively. This assent with the oxidation of four Cys residues that would course by the formation of at least one disulfide bridge (modification being responsible for enzyme inactivation). On the other hand, oxidation of Ga3PDHase seems to occur by a more complex mechanism. The formation of a disulfide bridge during oxidation of this enzyme cannot be discarded, as TRX-*h* can conspicuously restore enzyme activity. In fact, 1.0 ± 0.2 Cys was titrated in the oxidized enzyme and 3.1 ± 0.4 after reduction, which shows that two Cys lost the thiol form after oxidation, independently of the mechanism of oxidation. However, reversibility in the redox process for Ga3PDHase takes place only partially or depending on the degree of previous exposure to oxidants. When the oxidative treatment reaches a certain condition, it looks like if oxidation of Ga3PDHase thiol groups leads to the formation of sulfenic, sulfinic and sulfonic acids, as previously described for the enzyme from *Arabidopsis*[10]. Thus, reduction of Ga3PDHase by TRX-*h* (or GSH for this enzyme) would be possible if the disulfide bond is formed (perhaps also if oxidation renders sulfenic acid), but oxidation to higher extents would turn the process irreversible.

**Figure 6:**
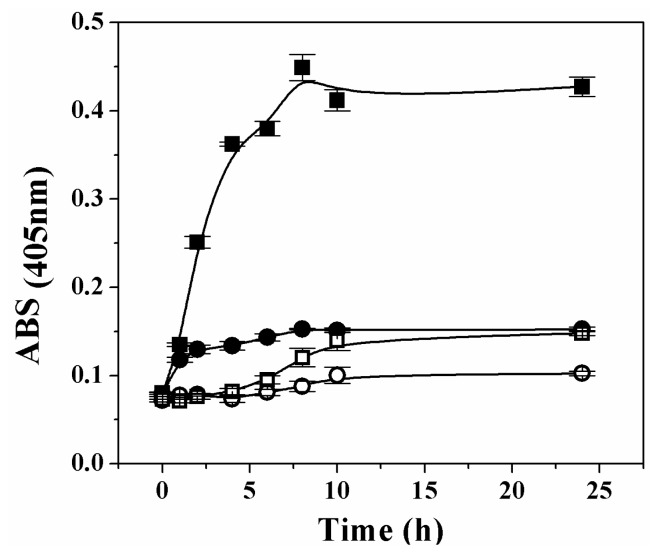
Over-oxidation of the triose-P DHases with H_2_O_2_. Time course turbidity followed by changes at 405 nm for solutions (0.6 mg/mL) of *np*-Ga3PDHase (open symbols) or Ga3PDHase (filled symbols) treated with none (circles) or with 1 mM H_2_O_2_ (squares). Error bars show deviation of determinations assayed as independent triplicates.

It has been reported that oxidative stress induces oligomerization and aggregation of human Ga3PDHase [23]. To check if over-oxidation provokes a similar effect on the plant recombinant triose-P DHases, we tested the oxidation of each enzyme by H_2_O_2_ at 37 °C and measured the turbidity during the 24 h treatment. Changes in the enzymes solubility (treated *in vitro* with or without H_2_O_2_) are represented in Figure 6. As shown, the absorbance for solutions of both enzymes remained with no significant change during a first period (~1 h) of exposure to the oxidant. However, the analysis of results in Figure 6 clearly indicates that after the first hour, further treatment of Ga3PDHase with H_2_O_2_ (but not that of *np*-Ga3PDhase) caused an increase in the turbidity. The precipitation of Ga3PDHase treated with H_2_O_2_ was also appreciated in an SDS-PAGE electrophoresis, in which resolution of the soluble and insoluble protein fractions (previously separated by centrifugation), showed a time dependent increase of the latter, with a concomitant decrease in the soluble protein (not shown).

## 3. Discussion

Herein we show that posttranslational redox modifications affect the activity of both cytosolic triose-P DHases, but in different ways. For both enzymes, the activity was slowed down by oxidizing agents related to ROS (H_2_O_2_), RNS (generated by SNP light exposure) and other physiological components (GSSG), but the reactivity of each DHase was different. Kinetic characterization of the oxidation reactions indicates that Ga3PDHase is more sensitive to oxidation (especially by H_2_O_2_) than *np*-Ga3PDHase, as the *k*” values obtained for the former were significantly higher (Table 1). Moreover, the addition of substrates attenuated inactivation by oxidation (Figure 3), in a way that agrees with the catalytic mechanism already described for each enzyme. As it was described, both enzymes have a cysteine residue (Cys^154^ in Ga3PDHase and Cys^298^ in np-Ga3PDHase from wheat) involved in the catalytic process which needs to be in the reduced state to form a thiohemiacetal intermediate with Ga3P [19]. In the case of Ga3PDHase, the protective effect exerted by Ga3P (when NAD^+^ is also present) suggests that Cys^154^ could be at least one of the residues being affected by oxidation. This fact was previously observed in the human (Cys^149^ in this case) [23] and the Arabidopsis ortholog enzymes [10].

**Figure 7:**
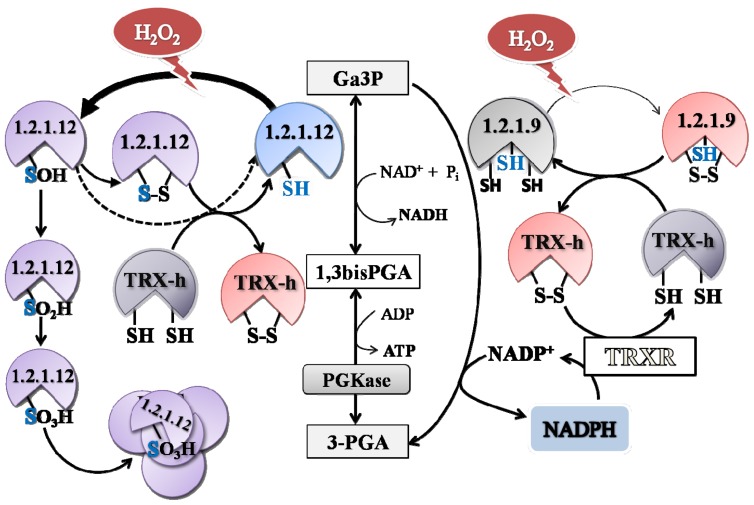
Scheme proposed for *np*-Ga3PDHase and Ga3PDHase oxidation. Redox regulation of Ga3PDHase (EC 1.2.1.12) could probably take place by routes involving one or more cystein residues. When two thiol groups are involved, a disulfide bridge would occur; but, as results partially support, some Cys would escape disulfide formation and become irreversibly oxidized by formation of sulfenic (SOH), sulfinic (SO_2_H) and sulfonic (SO_3_H) acid, depending on the oxidant involved, its concentration and the extent of the oxidation. Reduction of Ga3PDHase by TRX-*h* could be possible from the disulfide bridge (eventually also from sulfenic acid, dashed arrows), but the oxidation to sulfinic and sulfonic acids turns irreversible and could promote protein aggregation and precipitation. By the other hand, redox regulation of *np*-Ga3PDHase (EC 1.1.1.9) would occur by disulfide bond formation probably between two Cys residues different from the catalytic one (marked in blue), which can be reverted anytime by reduced TRX-*h.* The synthesis of NADPH by *np*-Ga3PDHase under the incidence of an oxidative situation, would favor the activity of antioxidant systems (like thioredoxin (TRX) which involves the thioredoxin reductase (TRXR) that maintains TRX-*h* in its reduced state). This would feedback NADPH production by maintaining the np-Ga3PDHase in its reduced (active) state.

Concerning *np*-Ga3PDHase, the fact that its oxidation was protected by addition of NADP^+^ (Figure 3a), also agrees with the mechanism proposed for the enzymatic reaction (where NADP^+^ is the first substrate interacting with the enzyme). However, the protective effect is not conclusive respect to if the catalytic Cys^298^ is actually affected by oxidation. In a previous report dealing with homology modeling of plant *np*-Ga3PDHases [22] it was shown that two cysteine residues (Cys^271^ and Cys^422^ in wheat *np*-Ga3PDHase), strictly conserved in the sequence of plant enzymes and absent in the protein from prokaryotes, could form a disulfide bond. From the results showed herein (Figures 1, 2 and 7) and the previous structural study, it could be thought the occurrence of a disulfide bond between Cys^271^ and Cys^422^ as a mechanism that protects the catalytic Cys^298^ from oxidation with the consequence of obstructing the substrates binding. It would also be proposed that after NADP^+^ binds to the enzyme, the proximity of that two Cys is modified as the structural change occurs, leaving them not correctly positioned to form the disulfide bridge. Altogether, the latter would allow a fine-tune regulation of *np*-Ga3PDHase activity under different cellular conditions, which in turn will modify Ga3P partitioning and levels of NADPH synthesis.

Interestingly, we also found that the lost of activity of the two cytosolic triose-P DHases caused by oxidation with H_2_O_2_ could be efficiently reverted by reduction with micromolar concentrations of TRX-*h*, a cytosolic isoform of this family of proteins that play a key role in cellular redox regulation (Figure 4) [24,25]. While the TRX-*h* dependent reduction of the *np*-Ga3PDHase was possible independently of how long is the previous exposure to the oxidant; reduction of Ga3PDHase by TRX-*h* was less effective when the exposure to oxidant increased the degree of inactivation of the enzyme (Figure 5). Our data support that redox regulation of Ga3PDHase and *np*-Ga3PDHase could occur physiologically. Results indicate that oxidation of each triose-P DHase takes place by different mechanisms, as proposed by the speculative scheme shown in Figure 7.

In agreement with previous reports for human Ga3PDHase [23], we observed that H_2_O_2_ over-oxidation of wheat recombinant Ga3PDHase induced protein aggregation and precipitation, an effect not observed in wheat recombinant *np*-Ga3PDHase (Figure 6). The latter particular property of the NAD^+^-dependent DHase (EC 1.2.1.12) would be functional in plant cells, probably associated with apoptosis after oxidative stress, a role found for the enzyme in human neurons [26,27]. Although irreversible protein oxidation triggers loss of native structure and functional activity, recent reports demonstrated that induced structural changes confer, to certain polypeptides, capacities to act as oxidative stress sensors. In this way, oxidized proteins become involved in several signal transduction routes (e.g., apoptosis activation), or they display “non-conventional” enzymatic activities (now acting as chaperones or transcriptional factors). In plants, as well as in mammals and some bacteria, an example of the latter is the 2Cys typical peroxiredoxin (2CysPRX) [28]. 2CysPRXs are peroxidases lacking prosthetic groups that mediate in the defense against oxidative stress by reducing H_2_O_2_ and alkyl hydroperoxides [29]. In addition to detoxifying peroxides, 2CysPRXs may be considered as peroxide sensors transmitting the presence of peroxides to upstream redox proteins; furthermore, the activities and properties of some Prx are regulated by overoxidation and nitrosylation [28]. As a whole, current information reported in plants agrees with that from animals, where it was demonstrated that Ga3PDHase is a multifunctional protein, with many roles besides its enzymatic activity in glycolysis [30]. Effectively, the enzyme from animals participates in nuclear events including gene transcription, RNA transport, DNA replication, and initiation of apoptotic cell death upon nitrosylation [26,27,30–33].

As a ROS, H_2_O_2_ has been given much attention during the past decades. A large amount of evidence has proven that H_2_O_2_ plays important roles as a signaling molecule in plants under unfriendly environmental conditions which include various biotic and abiotic stresses [20,34]. Also to some extent, an excess in H_2_O_2_ accumulation was shown that can lead to oxidative stress in plants, which then triggers cell death [35]. By its part, Ga3PDHase has emerged some years ago as a multifunctional enzyme involved in numerous cellular functions, and further investigations have yielded significant insight into its involvement in cellular response to oxidative stress and apoptosis in animal cells [30]. Regardless, much less is known about the non-enzymatic Ga3PDHase related functions in plants. After proteomic studies, it was proposed that Ga3PDHase could be involved in H_2_O_2_ perception [11,36]. Recently [37], it was also reported that cytosolic Ga3PDHase interact with the plasma membrane associated phospholipase D to translate the ROS H_2_O_2_ signal in Arabidopsis, in a way mediating the plant response to absicic acid and water deficits. Nevertheless, many studies need to be done to elucidate specific non-enzymatic roles played by the protein in plant cells.

Recently, Taniguchi and Miyake [38] revisited the plant redox-shuttling systems between chloroplast and cytosol. In their review, the authors remark the importance of fine control of the malate/oxaloacetate and triose-P/3PGA shuttle systems to maintain adequate levels of reductant and proper metabolic balance. The triose-P DHases under study are the cytosolic components of the enzymes involved in the triose-P/3PGA shuttle system, while the triose-P translocator (located in the inner envelope membrane) together with the Ga3PDHase (EC 1.2.1.13) and PGKase of the Calvin-Benson cycle are the chloroplast counterparts. Our results give new insights at the molecular level on the regulatory mechanism acting on this shuttle system. The regulation agrees with the function of *np*-Ga3PDHase as a cytosolic NADPH producing enzyme and its probable important role under oxidative conditions. In this sense, it has been reported that, in wheat and maize seedlings, the *np*-Ga3PDHase activity increase up to 2-fold after oxidative stress conditions imposed by methylviologen, as a consequence of a significant stability of the active enzyme (not due to mRNA induction) [4].

**Figure 8:**
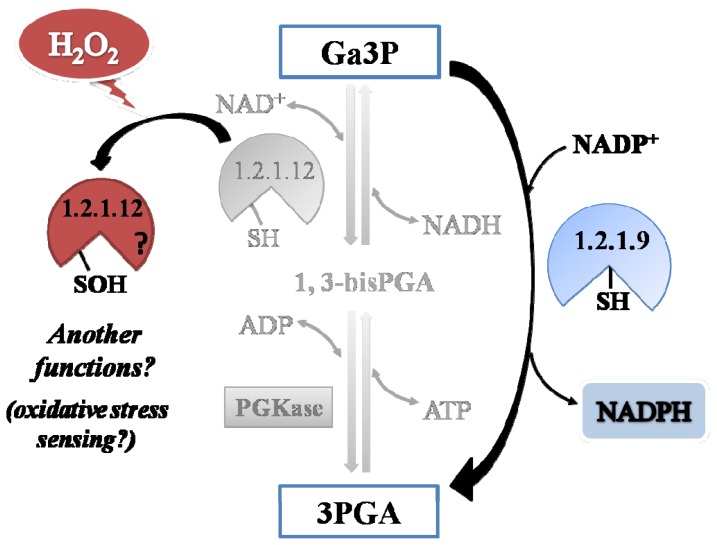
Partitioning of Ga3P during cytosolic glycolysis under the presence of H_2_O_2_. The picture represents that the presence of H_2_O_2_ triggers an alert signal, inducing oxidation of both triose-P DHases. The higher sensitivity to the oxidant of the Ga3PDHase enzyme (EC 1.2.1.12) provokes high oxidation states of critical Cys residues, resulting in a reduction of this pathway for the Ga3P oxidation (marked lighter). Conversely, the *np*-Ga3PDHase (EC 1.2.1.9) lower reactivity toward the oxidant makes the enzyme more stable, remaining active (considering also the reversion by TRX-*h*) and leading Ga3P to produce NADPH.

A comparative analysis of the kinetic constant values obtained for oxidation reactions of both Triose-P DHases let us to propose a redox scenario for Ga3P partitioning between these two pathways in the cytosol of plant cells (Figure 8). By this way, the presence of H_2_O_2_ would increase the metabolic flux of Ga3P through *np*-Ga3PDHase, with the consequence increment in the synthesis of NADPH (given that *np*-Ga3PDHase is significantly less reactive with H_2_O_2_ than Ga3PDHase). Enhanced NADPH synthesis would be beneficial for antioxidant systems coping with oxidative stress that depend on the reducing power to ameliorate the redox imbalance [21]. Furthermore, the process would feedback NADPH production maintaining *np*-Ga3PDHase in reduced state by TRX-*h* (which itself requires NADPH to be reduced by thioredoxin reductase). Therefore, the high reactivity of Ga3PDHase toward H_2_O_2_, would be related to its probable role as an oxidative sensor molecule.

Even when many studies on redox-regulated proteins and proteomic analyses have been reported [21,38], the knowledge of oxidative signal transduction pathways occurring in plants is far from complete. So far, our results are clearly in concordance with the physiological evidence already accounted on redox plant metabolism, and they allow assign a more detailed functioning of both triose-P DHases found in the cytosol of plant cells. Results obtained in this work, together with those published before, support the idea that *np*-Ga3PDHase could be involved in the response to oxidative stress to assure the synthesis of NADPH, while Ga3PDHase could be a sensor of the occurrence of the oxidative stress and, after oxidation, acts as a signaling molecule.

## 4. Experimental Section

### 4.1. Chemicals

Rabbit muscle aldolase, NAD^+^, NADP^+^, NADH, NADPH, Fru1,6bisP, 3PGA, diamide, H_2_O_2_, sodium nitroprusside (SNP), and DTT were purchased from Sigma-Aldrich (St. Louis, MO, USA). All other reagents were of the highest quality available.

### 4.2. Molecular Cloning, Expression and Purification of Recombinant Proteins

Recombinant wheat *np*-Ga3PDHase (NCBI accession N° DQ268762) was obtained from *Escherichia coli* BL21-CodonPlus^®^(DE3)-RIL (Novagen, Madison, WI, USA) cells transformed with (pRSETB/*TagapN*) as previously described [14]. The genes coding for wheat Ga3PDHase (*gapC*, NCBI accesion N° EF592180) and wheat TRX-*h* (*trxH*, NCBI N° AJ404845) were amplified from wheat (*Triticum aestivum*, *Tae*) leaves mRNA as it was previously described for *np*-Ga3PDHase [14] with the following specific oligonucleotides: *TaegapC* fow (5′-GGATCCGATGGGCAAGATTAAGATCGG-3′), *TaegapC* rev (5′-GAATTCTTACTTGGTGCTGTGCATGTG-3′), *TaetrxH* fow (5′-GGATCCGAT GGCGGCGTCGGCGGCGAC-3′) and *TaetrxH* rev (5′-AAGCTTTTACTGGGCCGCGTGTAGCC-3′).

For protein expression purposes the amplified genes were subcloned in the pRSETB expression vector (Invitrogen, Carlsbad, CA, USA), being the constructions (pRSETB/*TagapC*) and (pRSETB/*TatrxH*) used to transform competent *E. coli* BL21 CodonPlus^®^(DE3)-RIL cells. All proteins were expressed in LB medium and highly purified by ion metal affinity chromatography (IMAC) (Hi-Trap™ Chelating HP, GE-Healthcare) as previously described for *np*-Ga3PDHase [14]. Purified proteins were stored in elution buffer supplemented with 10 mM 2-mercaptoethanol and 10% (*v*/*v*) glycerol at −80 °C until use, conditions under which they were stable during at least 6 months.

### 4.3. Protein Methods

Total protein concentration was determined by the Bradford assay [39], using bovine serum albumin as standard. Protein electrophoresis under denatured conditions (SDS-PAGE) was performed as previously described by Laemmli [40].

### 4.4. Ga3PDHase and *np*-Ga3PDHase Activity Assay and Kinetics Studies

Ga3PDHase activity assay was performed following a protocol previously described with some modifications [13]. The reaction mixture (50 μL) contained (unless otherwise specified): 50 mM Tricine-NaOH pH 8.5, 1 mM NAD^+^, 10 mM sodium arsenate, 0.4 units aldolase (rabbit muscle), 1.2 mM fructose-1,6-bisphosphate (Fru1,6bisP), and an adequate quantity of enzyme.

The activity of *np*-Ga3PDHase was assayed as previously described [41]. The reaction mixture (50 μL) contained (unless otherwise specified): 50 mM Tricine-NaOH pH 8.5, 0.2 mM NADP^+^, 0.4 units aldolase (rabbit muscle), 1.2 mM Fru1,6bisP, and an adequate quantity of enzyme.

In both cases the reaction was performed at 30 μC and started with the addition of Fru1,6bisP. NADH or NADPH generation was monitored spectrophotometrically at 340 nm. One unit (U) is defined as the amount of enzyme that catalyzes the formation of 1 μmol NAD(P)H per minute under the specified assay conditions.

For enzyme kinetics, saturation curves were performed by assaying the respective enzyme activity at saturating level of the fixed substrate and different concentrations of the variable substrate. Values of *S*_0.5_, *V*_max_ and Hill numbers (*n*_H_) were obtained fitting the experimental data to the generalized Hill equation using the Levenberg-Marquardt nonlinear least-squares algorithm provided by the computer program Origin™ 7.0 All kinetic parameters are the mean of at least three determinations and were reproducible within at least ±10%.

### 4.5. Oxidation Assay

Purified Ga3PDHase and *np*-Ga3PDHase were desalted using Microcon spin columns (Millipore) in 100 mM Tris-HCl pH 8.0, 0.1 mM EDTA as a previous procedure to remove 2-mercaptoethanol (similar results were obtained at pH values between 7 and 8). For oxidation, 0.024 μg/μL of protein were incubated in the presence of different concentrations of either diamide, H_2_O_2_, SNP or GSSG, in buffer 100 mM Tris-HCl pH 8.0, 0.1 mM EDTA at 25 °C. The incubation with SNP was carried out with direct environment light to induce photolytic decomposition of the reagent and generate nitric oxide and other reactive nitrogen species. After different incubation times, aliquots were withdrawn, conveniently diluted and assayed for enzyme activity as above described.

### 4.6. Kinetic Analysis of Oxidation Reactions

The oxidation rate for Ga3PDHase and *np*-Ga3PDHase, was followed by withdrawing samples for assay of activity at different time intervals after the mixing of the respective enzyme with at least six different concentrations of each oxidant. Experimental data were plotted as log remaining activity against the oxidation reaction time (min or s). Percentage of remaining activity was calculated taken as 100% the activity of the specific enzyme incubated under the same conditions but in the absence of oxidants. The first order rate constant (*k*_app_) for each oxidant concentration was calculated by fitting the curves to the equation: log relative activity = 2 − *k*_app_*t, with the software Origin™ 7.0. Plots of *k*_app_ values *vs.* variable oxidant concentrations allowed identifying if the mechanism of inactivation coursed via one-step (the plot was linear) or two-step (the plot showed hyperbolic) model, as proposed by Kitz and Wilson [16] for the irreversible inhibition of enzymes:

(1) $$E+I\overset{K_{\text{i}}}{⇄}EI\overset{k_{\text{i}}}{\to }E-I\;\;\;k_{\text{app}}=k_{\text{i}}*I/(K_{\text{i}}+I)\;\;\;k^{\prime \prime }=k_{\text{i}}/K_{\text{i}}$$

For this mechanism, *k*_i_ defines the maximum rate of inactivation that is achieved at an infinite concentration of inactivator, *K*_i_ describes the concentration of inhibitor yielding a rate of inactivation equal to half *k*_i_, and *k*” = *k*_i_/*K*_i_ is the second-order rate, which is considered to be the best measure of relative inactivator potency. When inactivation courses in one step *k*_app_ = *k*”*(*I*) and the value of *k*” is calculated directly by linear regression.

To calculate the dissociation constant (*K*_d_) for the binding of the substrate to the respective triose-P PDHase, inactivation of the enzyme at a fixed concentration of H_2_O_2_ was afforded by different concentrations of Ga3P (in the presence on NAD^+^ 1 mM) for the Ga3PDHase, or different concentrations of NADP^+^ in the case of the *np*-Ga3PDHase. Inactivation kinetic data obtained in the different conditions were plotted according to Mildvan and Leigh [42], using the equation:

(2) $$1/k{’}_{\text{app}}=1/k’+a/K_{\text{d}}*k’$$

where *k*’ is the observed first-order rate constant of inactivation in the absence of the protective agent, and *k*’_app_ the apparent inactivation constant obtained at different concentrations of *a*, the compound affording protection.

### 4.7. Reduction Assay

Ga3PDHase and *np*-Ga3PDHase oxidized as specified above were diluted and extensively desalted/re-concentrated to remove the oxidant in 50 mM Tris-HCl pH 8.0 using Microcon spin columns (Millipore). The oxidized enzymes were incubated with DTT or reduced TRX-*h* in buffer 50 mM Tris-HCl pH 8.0 (similar results were obtained in the pH range 7–8). After different incubation times, aliquots were withdrawn from the incubation media and assayed for enzyme activity. Previous to use, TRX-*h* was reduced by incubation in buffer 100 mM Tris-HCl pH 8.0, 5% (*v*/*v*) glycerol, 0.1 mM EDTA, and 0.5 mM DTT during 30 min at 25 °C.

### 4.8. *In Vitro* Turbidity Assay to Detect Protein Precipitation by Oxidation

Recombinant Ga3PDHase or *np*-Ga3PDHase (0.6 mg/mL) were treated with H_2_O_2_ in buffer 50 mM Tris-HCl pH 8.0, 150 mM NaCl, 1 mM EDTA and 5% (*v*/*v*) glycerol at 37 °C and aliquots were withdrawn at different times ranging from 0 to 24 h. Turbidity of samples (derived from aggregated oxidized proteins) was measured following the absorbance at 405 nm in a microplate reader [23]. Immediately after turbidity measurement, the samples were centrifuged (14,000 rpm, 20 min) to separate soluble and insoluble fractions. All samples were subjected to SDS-PAGE [40].

### 4.9. Colorimetric Reduced Thiol Determination

Determination of reduced thiol residues in both recombinant Ga3PDHases under reduced and oxidized state was assayed as previously described by Ellman [43]. Ga3PDHase (12 μM) was oxidized with 1 mM H_2_O_2_ for 5 min and *np*-Ga3PDHase (18 μM) was oxidized with 3 mM H_2_O_2_ for 20 min in buffer 100 mM Tris-HCl pH 8.0, 150 mM NaCl at 25 °C. In both cases the reduced state was achieved by incubation with 1 mM DTT for 20 min. After treatments, proteins were desalted by centrifugation using Microcon spin columns (Millipore). For determination of reduced thiol residues in the native proteins an aliquot was incubated 30 min with 2 mM DTNB (5,5′-ditiobis(2-benzoate)) in buffer 100 mM Tris-HCl pH 8.0, 150 mM NaCl at 25 °C. Color development was measured at 405 nm in a microplate reader.

## 5. Conclusions

In this work, we performed a comparative analysis of posttranslational redox regulation between enzymes acting in parallel on the glycolytic pathway. The results obtained at the proteins molecular level, led us to conclude that Ga3P partitioning between Ga3PDHase and *np*-Ga3PDHase would change under different metabolic scenarios, with a different balance in energy or reducing power synthesis. The outcomes indicate that oxidative conditions would favor the Ga3P metabolism via *np*-Ga3PDHase, increasing the synthesis of reducing power, which in turn would favor antioxidant systems action to cope with oxidative stress.

## Supplementary Information



## Figures and Tables

**Table 1:** Kinetic parameters for the oxidation of recombinant *np*-Ga3PDHase and Ga3PDHase with H_2_O_2_, SNP or GSSG. Values were calculated using the irreversible inhibitor model as described under the Experimental Section. Oxidation of both enzymes by SNP and GSSG occurred in two-step (T; with formation of an intermediary complex EI) while H_2_O_2_ oxidation mechanism occurred in one-step (O, with no appreciable formation of an intermediary complex).

Oxidant	Mechanism	*k*_i_ (min^−1^)	*K*_i_ (mM)	*k*” (M^−1^s^−1^)
*np*-Ga3PDHase				

H_2_O_2_	O	nd *	nd	1.9
SNP	T	0.036	0.6	1.0
GSSG	T	0.011	2.50	0.07
Ga3PDHase				

H_2_O_2_	O	nd	nd	115.0
SNP	T	1.3	4.8	4.5
GSSG	T	0.13	12.30	0.17

* nd: not determined.
