# Supplementary material for: A Differential Redox Regulation of the Pathways Metabolizing Glyceraldehyde-3-Phosphate Tunes the Production of Reducing Power in the Cytosol of Plant Cells
Source: Int J Mol Sci. 2013 Apr 12;14(4):8073–92. doi: 10.3390/ijms14048073 (PMC3645732; doi:10.3390/ijms14048073)
Supplement: Supplementary file 1 [file ijms-14-08073-s001.pdf]

Supplementary Information

Figure S1. Electrophoretic profiles (Coomassie blue stained 12% SDS-PAGE) for the expression and purification of recombinant *np*-Ga3PDHase (**a**) and Ga3PDHase (**b**). (**a**) Molecular mass markers (lane 1), soluble extract from *Escherichia coli* BL21-CodonPlus®(DE3)-RIL cells transformed with (pRSETB/*TagapN*) (the plasmid construct to express wheat *np*-Ga3PDHase) after 5 h induction with 0.5 mM IPTG (lane 2), unbound proteins from IMAC purification (lane 3), IMAC wash step with 80 mM imidazole (lane 4), elution of IMAC bound *np*-Ga3PDHase with 300 mM imidazole (lanes 5 and 6); (**b**) Soluble extract from *Escherichia coli* BL21-CodonPlus®(DE3)-RIL cells transformed with (pRSETB/*TagapC*) (the plasmid construct to express wheat Ga3PDHase) after 5 h induction with 0.5 mM IPTG (lane 1), unbound proteins from IMAC purification (lane 2), IMAC wash step with 40 mM imidazole (lane 3), IMAC wash step with 80 mM imidazole (lane 4), molecular mass markers (lane 5), elution of IMAC bound Ga3PDHase with 300 mM imidazole (lanes 6 and 7).

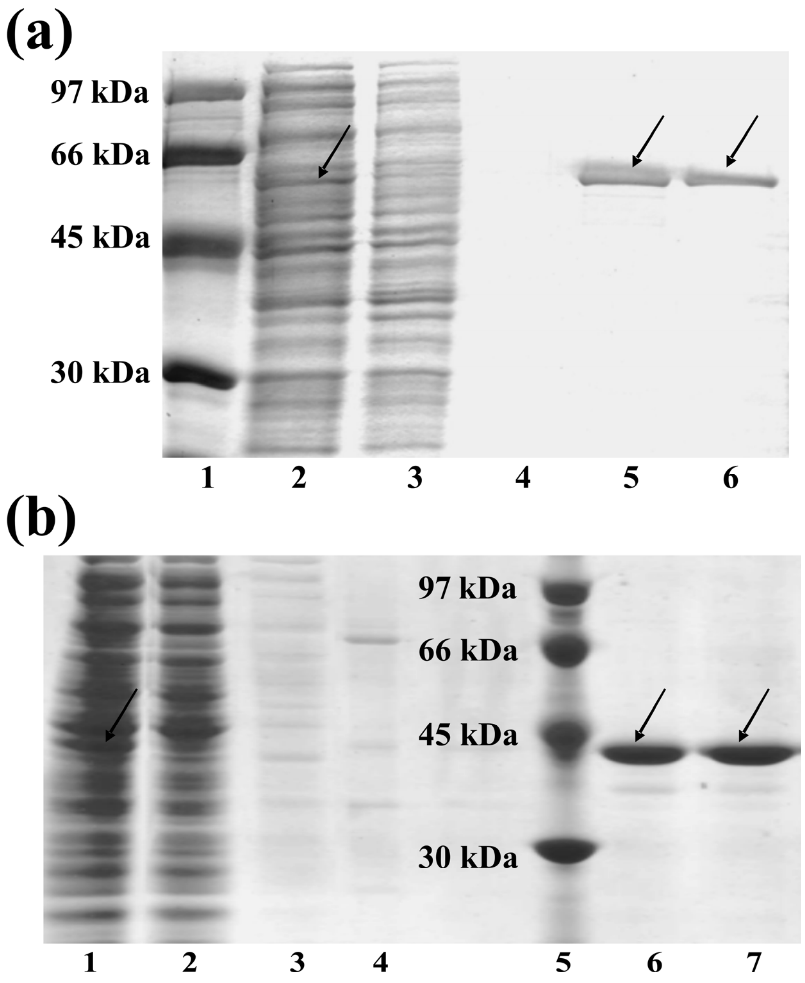

Figure S2. Molecular mass determination of recombinant Ga3PDHase on Superdex 200. Size exclusion chromatography was performed using a Tricorn5/200 column and a gel filtration calibration kit (both from GE healthcare). Molecular mass (MM) standards include: thyroglobulin (669 kDa), ferritin (440 kDa), aldolase (158 kDa), conalbumin (75 kDa), and ovoalbumin (44 kDa).

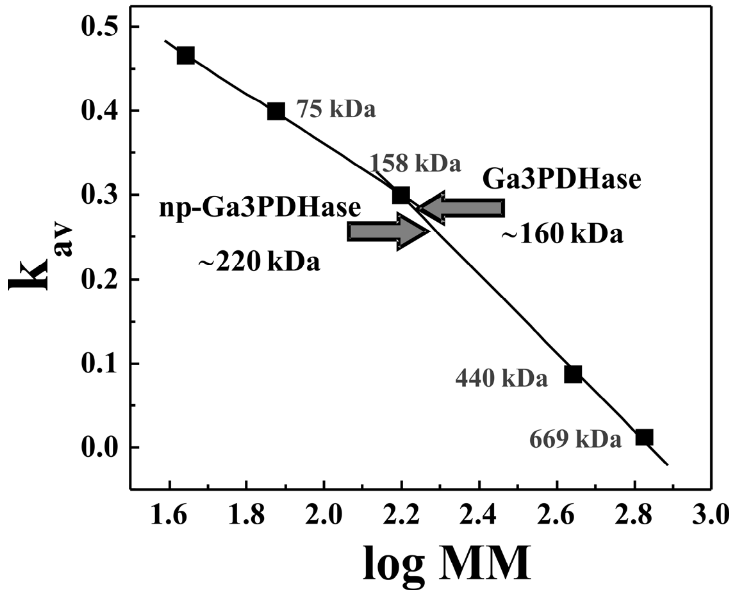

© 2013 by the authors; licensee MDPI, Basel, Switzerland. This article is an open access article distributed under the terms and conditions of the Creative Commons Attribution license (http://creativecommons.org/licenses/by/3.0/).
